# Creatine Kinase and Mortality in Peritoneal Dialysis

**DOI:** 10.3389/fcvm.2022.855891

**Published:** 2022-05-10

**Authors:** Xianfeng Wu, Lei Zhou, Xiaojiang Zhan, Yueqiang Wen, Xiaoyang Wang, Xiaoran Feng, Niansong Wang, Fenfen Peng, Junnan Wu

**Affiliations:** ^1^Department of Nephrology, Shanghai Jiao Tong University Affiliated Sixth People’s Hospital, Shanghai, China; ^2^Clinical Research Center for Chronic Kidney Disease, Shanghai Jiao Tong University Affiliated Sixth People’s Hospital, Shanghai, China; ^3^Department of Nephrology, Zhejiang University Medical College Affiliated Sir Run Run Shaw Hospital, Hangzhou, China; ^4^Department of Nephrology, The First Affiliated Hospital of Nanchang University, Nanchang, China; ^5^Department of Nephrology, The Second Affiliated Hospital of Guangzhou Medical University, Guangzhou, China; ^6^Department of Nephrology, The First Affiliated Hospital of Zhengzhou University, Zhengzhou, China; ^7^Department of Nephrology, Jiujiang No.1 People’s Hospital, Jiujiang, China; ^8^Department of Nephrology, Zhujiang Hospital of Southern Medical University, Guangzhou, China

**Keywords:** creatine kinase, mortality, cardiovascular mortality, peritoneal dialysis, prognosis

## Abstract

**Background:**

The association between serum creatine kinase and mortality in patients with peritoneal dialysis (PD) remained unknown.

**Methods:**

We retrospectively collected data on 3,446 incident patients with from five PD centers in China between 1 January 2005 and 31 May 2020. Creatine kinase was collected 1 week before the start of PD. We examined the association between creatine kinase and mortality using Cox proportional hazards model.

**Results:**

The median creatine kinase was 113 (range, 1.22–4,574) IU/L. With a median follow-up of 39.5 (range, 3.1–181.5) months, 763 (22.1%) all-cause deaths occurred, including 384 (11.1%) cardiovascular deaths. As compared with a creatine kinase of 111–179 IU/L (reference range), a higher creatine kinase (>179 IU/L) was associated with increased risks of all-cause mortality [hazards ratio (HR), 1.72; 95% CI, 1.35–2.00; *E*-value = 2.83] and cardiovascular mortality (HR, 1.44; 95% CI, 1.05–1.98; *E*-value = 2.24). As compared with the reference range, a lower creatine kinase (<111 IU/L) was associated with increased risks of all-cause mortality (HR, 1.40; 95% CI, 1.12–1.76; *E*-value = 2.15) and cardiovascular mortality (HR, 1.45; 95% CI, 1.08–1.94; *E*-value = 2.26). Interaction between creatine kinase and no hyperlipidemia (*p* = 0.034 for interaction) was observed.

**Conclusion:**

A creatine kinase before the start of PD between 111 and 179 IU/L was associated with a lower risk of death than a higher or lower creatine kinase, resulting in a U-shaped association curve.

## Introduction

Creatine kinase is an important enzyme that consumes adenosine triphosphate rapidly (1). Serum creatine kinase levels are higher in healthy men and associated with muscle mass and body mass index (2–4). Serum creatine kinase activity is detected in myocardial infarction, rhabdomyolysis, myositis, and muscle dystrophy (5). Several previous studies reported that creatine kinase was positively associated with blood pressure (6, 7) and was associated with the failure of antihypertensive therapy in the general population (8). A 12-year population-based cohort study in Japan showed that elevated serum creatine kinase levels were associated with a moderately increased risk for myocardial infarction (9). More importantly, previous studies reported that high serum creatine kinase levels were associated with increased mortality in patients with rhabdomyolysis, traumatic injuries, hantaviruses, and genetic myopathies (10–13). Notably, there to date was no study regarding the association between serum creatine kinase and mortality in patients with peritoneal dialysis (PD).

A recent study showed that a low serum creatine kinase level was associated with an increased risk of death in the non-dialysis chronic kidney disease (CKD) population (5), which was inconsistent with those findings above. Early study found that elevated creatine kinase had been reported in dialysis patients compared with the general population (14). Thus, based on these findings earlier, we wondered whether elevated or lowered levels of creatine kinase were associated with an increased risk of mortality in patients with dialysis. In this study, we examined the association between creatine kinase and mortality in patients on continuous ambulatory peritoneal dialysis (CAPD).

## Materials and Methods

### Study Design and Participants

We conducted a retrospective study that included 3,566 incident patients with CAPD from five PD centers in China between 1 January 2005 and 31 May 2020. To evaluate the association between creatine kinase 1 week before the start of PD and mortality in the real-world setting, we only excluded patients aged <18 years and those with <3 months of the follow-up. Plus, although no vigorous exercise was recorded in medical records, patients with creatine kinase ≥5,000 IU/L were excluded according to rhabdomyolysis, which is defined as levels of five times above the upper limit of normal serum creatine kinase (1,000 IU/L) (15). Baseline data were collected 1 week before the start of PD, representing a severe uremic status. Therefore, it was challenging for patients to perform excessive physical activity, and we did not evaluate physical activity in our study. The data were anonymous, and the need for informed consent was waived. The study protocol complied with the Declaration of Helsinki and had full approval from each Clinical Research Ethics Committee.

### Data Collection and Follow-Up

We respectively collected demographic data, comorbidities, medication use, and laboratory data 1 week (5.3 ± 1.2 days) before the start of PD, including age at study entry, sex, body mass index, current smoker, current alcohol use, systolic blood pressure, diastolic blood pressure, comorbidities [diabetes mellitus, hypertension, prior cardiovascular disease, hyperlipidemia, chronic obstructive pulmonary disease (COPD), and gastrointestinal bleeding], medication use [calcium antagonist, beta-blockers, angiotensin-converting enzyme inhibitors/angiotensin II receptor blockers (ACEI or ARBs), diuretics, and statins], and laboratory measurements [serum creatine kinase, hemoglobin, albumin, estimated glomerular filtration rate (eGFR), high-density lipoprotein (HDL), low-density lipoprotein (LDL), and serum sodium].

Our primary outcome measure was all-cause and cardiovascular mortality. Details for the CAPD follow-up were previously described elsewhere (16). The follow-up period was from the start of PD to the date of death, transfer to hemodialysis, receiving renal transplantation, transfer to other dialysis centers, loss of follow-up, or 31 May 2020. Patients who were lost to follow-up were censored at the date of the last examination.

### Statistical Analysis

Differences in the baseline characteristics among the study population in the different categories of creatine kinase were compared using the chi-square test for categorical variables and ANOVA for continuous variables. We used restricted-cubic-spline plots to explore the shape of the association between creatine kinase and mortality, fitting a restricted-cubic-spline function with four knots (at the 25th, 50th, 75th, and 95th percentiles) (17).

Based on our restricted-cubic-spline plots for the primary outcome, we selected a level of 111–179 IU/L as the reference category for creatine kinase. Cumulative all-cause and cardiovascular mortality were analyzed using the Kaplan–Meier failure function. We performed a multivariable Cox proportional hazards model in order to determine the association between creatine kinase and all-cause and cardiovascular mortality, using four sequential models. Model 1 was adjusted for age, sex, body mass index, current smoker (yes or no), current alcohol use (yes or no), and systolic blood pressure. Model 2 included model 1 and diabetes mellitus (yes or no), hypertension (yes or no), prior cardiovascular events (yes or no), COPD, gastrointestinal bleeding, and hyperlipidemia. Model 3 included model 2 and taking calcium antagonist (yes or no), beta-blocker (yes or no), ACE inhibitor or ARB (yes or no), diuretics (yes or no), and statins (yes or no). Model 4 included model 3 and hemoglobin, albumin, eGFR, HDL, LDL, and serum sodium.

We tested for interactions of age, sex, diabetes mellitus, hypertension, prior cardiovascular disease, and hyperlipidemia. We explored the potential influence of unmeasured confounders on our risk estimates using *E*-value analysis to determine how solid and imbalanced a confounding effect would need to be to alter the direction of findings (18). To minimize the potential for reverse causation, we conducted analyses that excluded patients with prior cardiovascular disease or those deaths in the first 2 years of follow-up. When considering a transfer to hemodialysis, receiving renal transplantation, transfer to other centers, and loss of follow-up as competing risks for all-cause mortality, we further analyzed the association between creatine kinase and all-cause mortality using the Gray test. Missing data for serum creatine kinase (*n* = 97) or any other explanatory variables (*n* = 143) at the start of PD were replaced by the most recent available values by checking patients’ medical records of receiving the first PD procedure. All the analyses were conducted with Stata 15.1. statistical software (StataCorp, College Station, TX, United States).

## Results

### Baseline Characteristics

We excluded 55 patients aged <18 years, 62 patients with less than 3 months of follow-up, and three patients with creatine kinase ≥5,000 IU/L. Thus, 3,446 eligible patients were finally included in this study.

**FIGURE 1:**
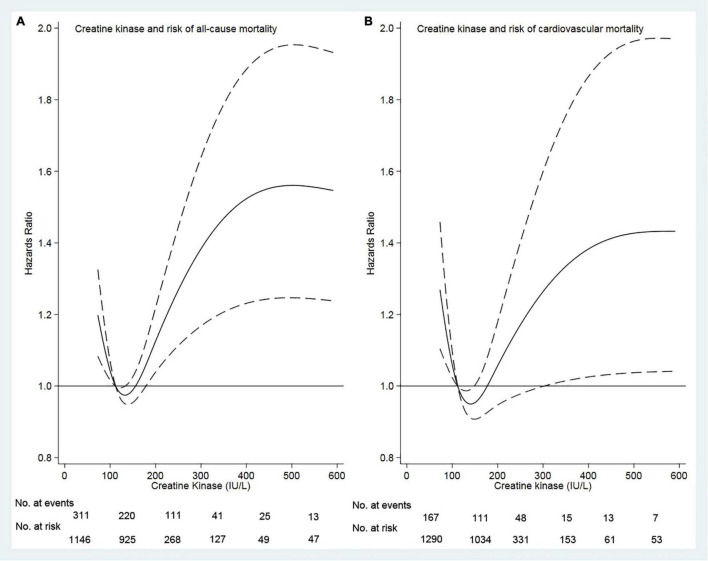
Association of serum creatine kinase with risk of mortality. Panel **(A)** showed a restricted-cubic-spline plot of the association between creatine kinase and the all-cause mortality. Panel **(B)** showed a restricted cubic–spline plot of the association of creatine kinase and cardiovascular mortality. All the plots were adjusted for age, sex, body mass index, current smoker, current alcohol use, systolic blood pressure, comorbidities, medication use, and lab measurements. Dashed lines indicate 95% CIs. The median creatine kinase (113 IU/L) was the reference standard, indicated by the red line.

Of 3,446 patients with a mean age of 49.6 years, 1,796 (52.1%) were male sex, 662 (19.2%) had diabetes mellitus, 2,415 (70.1%) had hypertension, 368 (10.7%) had a history of cardiovascular disease, and 597 (17.3%) had hyperlipidemia. The median creatine kinase was 113 (range, 1.22–4,574) IU/L. Based on our restricted-cubic-spline plots for the primary outcome, we selected a level of 111–179 IU/L as the reference category for creatine kinase (Figure 1). The baseline characteristics of patients according to creatine kinase were shown in Table 1. Compared with the moderate group, the high group was more likely to be female and diabetes mellitus, but less likely to be current smoker and alcohol use. In contrast, the low group was more likely male gender and current alcohol use but less likely to be taking ACEI or ARB.

**TABLE 1:** Baseline characteristics of the study patients, according to creatine kinase.

	Creatine kinase				
Characteristics	All levels	Low (<111 IU/L)	Moderate (111–179 IU/L)	High (>179 IU/L)	*P*-value
Proportion of participants, n	3,446	1,679	800	967	
Creatine kinase (IU/L)	113 (73–195)	72 (57–90)	136 (123–153)	292 (225–478)	
Age (years)	49.6 ± 14.4	50.0 ± 14.5	48.7 ± 14.2	49.7 ± 14.6	0.136
Male sex, n (%)	1,796 (52.1%)	1,171 (69.7%)	437 (54.6%)	188 (19.4%)	<0.001
Body mass index (kg/m^2^)	22.4 ± 3.3	22.3 ± 3.3	22.3 ± 3.2	22.4 ± 3.3	0.71
Current smoker, n (%)	349 (10.1%)	233 (13.9%)	99 (12.4%)	17 (1.8%)	<0.001
Current alcohol use, n (%)	128 (3.7%)	85 (5.1%)	37 (4.6%)	6 (0.6%)	<0.001
Systolic blood pressure (mmHg)	147.6 ± 22.8	146.8 ± 22.9	148.3 ± 22.1	148.4 ± 23.0	0.134
Diastolic blood pressure (mmHg)	87.1 ± 14.0	86.6 ± 13.5	87.7 ± 14.1	87.6 ± 14.8	0.087
**Comorbidities, n (%)**					
Diabetes mellitus	662 (19.2%)	296 (17.6%)	142 (17.8%)	224 (23.2%)	0.001
Hypertension	2,415 (70.1%)	1,193 (71.1%)	537 (67.1%)	685 (70.8%)	0.113
Prior cardiovascular disease	368 (10.7%)	200 (11.9%)	71 (8.9%)	97 (10.0%)	0.054
COPD	30 (0.9%)	14 (0.8%)	6 (0.8%)	10 (1.0%)	0.794
Gastrointestinal bleeding	95 (2.8%)	51 (3.0%)	20 (2.5%)	24 (2.5%)	0.618
Hyperlipidemia	597 (17.3%)	298 (17.7%)	133 (16.6%)	166 (17.2%)	0.778
**Medication use, n (%)**					
Calcium antagonist	2,202 (63.9%)	1,062 (63.3%)	506 (63.3%)	634 (65.6%)	0.441
Beta-blocker	1,305 (37.9%)	637 (37.9%)	291 (36.4%)	377 (39.0%)	0.528
ACE inhibitor or ARB	946 (27.5%)	418 (24.9%)	233 (29.1%)	295 (30.5%)	0.004
Diuretics	547 (15.9%)	274 (16.3%)	137 (17.1%)	136 (14.1%)	0.169
Statins	510 (14.8%)	261 (15.5%)	105 (13.1%)	144 (14.9%)	0.283
**Laboratory measurements**					
Hemoglobin (g/L)	87.7 ± 19.8	88.0 ± 19.7	88.2 ± 19.4	86.7 ± 20.3	0.203
Albumin (g/L)	34.5 ± 5.3	34.7 ± 5.2	34.6 ± 5.1	34.1 ± 5.5	0.014
eGFR (mL/min × 1.73 m^2^)	7.1 ± 3.8	7.7 ± 3.9	6.9 ± 3.4	6.5 ± 3.7	<0.001
HDL (mEq/L)	1.14 ± 0.39	1.09 ± 0.36	1.16 ± 0.40	1.19 ± 0.43	<0.001
LDL (mEq/L)	2.56 ± 0.89	2.48 ± 0.86	2.59 ± 0.81	2.69 ± 0.97	<0.001
Serum sodium (mEq/L)	140.1 ± 3.8	140.1 ± 3.7	140.1 ± 3.8	140.0 ± 4.0	0.769

*COPD, chronic obstructive pulmonary disease; ACEI, angiotensin-converting enzyme inhibitor; ARB, angiotensin receptor blocker; eGFR, estimated glomerular filtration rate; HDL, high-density lipoprotein; LDL, low-density lipoprotein.*

### Creatine Kinase and Mortality

During the median of 39.5 (range, 3.1–181.5) months of follow-up, 763 (22.1%) patients died, 466 (13.5%) patients transferred to hemodialysis, 229 (6.6%) patients received renal transplantation, 434 (12.6%) patients transferred to other dialysis centers, and 58 (1.7%) patients had been the loss of follow-up. Of 763 deaths, 384 (50.3%) deaths were due to cardiovascular disease, 135 (17.8%) deaths due to infectious disease, 72 (9.4%) deaths due to gastrointestinal bleeding, 14 (1.8%) deaths due to malignancy, 66 (8.7%) deaths due to other reasons, and 92 (12.1%) deaths due to unknown reasons. Deaths occurred in 370 (56.5/1,000 person-years), 134 (40.7/1,000 person-years), and 259 (63.2/1,000 person-years) patients in those <111, 111–179, and >179 IU/L patients, respectively (Table 2).

**TABLE 2:** The incidence rate of death according to creatine kinase.

	Creatine kinase			
Outcomes	All levels	Low (<111 IU/L)	Moderate (111–179 IU/L)	High (>179 IU/L)
Proportion of participants, n	3,446	1,679	800	967
Person-years	13,946.7	6,551.6	3,294.8	4,100.4
**All-cause mortality**				
Events, n	763	370	134	259
Events, per 1,000 person-years	54.7	56.5	40.7	63.2
**Cardiovascular mortality**				
Events, n	384	198	67	119
Events, per 1,000 person-years	27.5	30.2	20.3	29.0

*The incidence rate was calculated by dividing the proportion of events by the total effective observation time in the risk, which is converted to the number of episodes per 1,000 years.*

**FIGURE 2:**
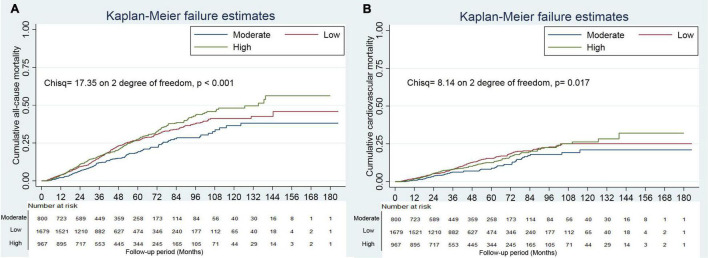
Cumulative mortality incidence by categories of creatine kinase. Panel **(A)** showed cumulative all-cause mortality by categories of creatine kinase. Panel **(B)** showed cumulative cardiovascular mortality by categories of creatine kinase.

Survival analyses showed that a creatine kinase of 111–179 IU/L had the lowest cumulative all-cause and cardiovascular mortality (Figure 2). As compared with a creatine kinase of 111–179 IU/L (the reference category), a creatine kinase of >179 IU/L was associated with increased risks of all-cause mortality [hazards ratio (HR), 1.72; 95% CI, 1.35–2.00; *E*-value = 2.83] and cardiovascular mortality (HR, 1.44; 95% CI, 1.05–1.98; *E*-value = 2.24) on multivariable analysis (Tables 3, 4 and Figure 1). Plus, as compared with the reference range, a lower serum creatine kinase (<111 IU/L) was also associated with increased risks of all-cause mortality (HR, 1.40; 95% CI, 1.12–1.76; *E*-value = 2.15) and cardiovascular mortality (HR, 1.45; 95% CI, 1.08 to 1.94; *E*-value = 2.26) on multivariable analysis (Tables 3, 4 and Figure 1).

**TABLE 3:** Association of creatine kinase with all-cause mortality.

	Creatine kinase		
	Low (<111 IU/L)	Moderate (111–179 IU/L)	High (>179 IU/L)
All-cause mortality, n (%)	370 (22.0%)	134 (16.8%)	259 (26.8%)
**Analysis, hazards ratio (95%CI)**			
Univariate analysis	1.40 (1.13–1.75)	1.0	1.81 (1.44–2.30)
**Multivariate analysis**			
Model 1	1.44 (1.15–1.80)	1.0	1.73 (1.36–2.20)
Model 2	1.42 (1.14–1.78)	1.0	1.72 (1.36–2.20)
Model 3	1.40 (1.12–1.76)	1.0	1.73 (1.35–2.00)
Model 4	1.40 (1.12–1.76)	1.0	1.72 (1.35–2.00)
Analysis excluding prior cardiovascular disease*	1.32 (1.04–1.68)	1.0	1.70 (1.31–2.21)
Analysis excluding deaths year 1 and 2*	1.31 (1.04–1.70)	1.0	1.74 (1.32–2.31)

*Model 1 included age, sex, body mass index, alcohol intake, current smoking, and systolic blood pressure. Model 2 included variables in model 1 and comorbidities. Model 3 included variables in model 2 and medication use. Model 4 included variables in model 3 and laboratory measurements.*

*Adjusted for variables included in model 4.

**TABLE 4:** Association of creatine kinase with cardiovascular mortality.

	Creatine kinase		
	Low (<111 IU/L)	Moderate (111–179 IU/L)	High (>179 IU/L)
Cardiovascular mortality, n (%)	198 (11.8%)	67 (8.4%)	119 (12.3%)
**Analysis, hazards ratio (95%CI)**			
Univariate analysis	1.46 (1.09–1.96)	1.0	1.54 (1.12–2.10)
**Multivariate analysis**			
Model 1	1.47 (1.10–1.98)	1.0	1.46 (1.06–2.00)
Model 2	1.45 (1.08–1.95)	1.0	1.44 (1.05–1.98)
Model 3	1.44 (1.08–1.94)	1.0	1.44 (1.05–1.98)
Model 4	1.45 (1.08–1.94)	1.0	1.44 (1.05–1.98)
Analysis excluding prior cardiovascular disease*	1.40 (1.04–1.88)	1.0	1.46 (1.06–2.00)
Analysis excluding deaths year 1 and 2*	1.42 (1.09–2.05)	1.0	1.55 (1.05–2.30)

*Model 1 included age, sex, body mass index, alcohol intake, current smoking, and systolic blood pressure. Model 2 included variables in model 1 and comorbidities. Model 3 included variables in model 2 and medication use. Model 4 included variables in model 3 and laboratory measurements.*

*Adjusted for variables included in model 4.

### Subgroup Analysis

No hyperlipidemia modified the association between a low creatine kinase and all-cause mortality (*p* = 0.034 for interaction, Table 5). In further analysis, significantly increased risk was observed in patients with no hyperlipidemia and a creatine kinase of <111 IU/L (all-cause mortality: HR, 1.56; 95% CI, 1.22–2.01), whereas there was no significant association among those with hyperlipidemia. Plus, no hyperlipidemia also modified the association between a low creatine kinase and cardiovascular mortality (*p* = 0.023 for interaction, Table 6). There were no other significant subgroup interactions (Tables 5, 6).

**TABLE 5:** Association of creatine kinase with all-cause mortality in subgroups.

	Creatine kinase				
	Event, n (%)	Low (<111 IU/L)	Moderate (111–179 IU/L)	High (>179 IU/L)	P-interaction
All-cause mortality					
<65 years	462 (16.1%)	1.25 (0.95–1.64)	1.0	1.52 (1.14–2.03)	0.145
≥65 years	301 (51.7%)	1.67 (1.06–2.63)	1.0	2.63 (1.58–4.40)	
Male	291 (16.2%)	1.30 (0.91–1.86)	1.0	2.12 (1.46–3.08)	0.875
Female	472 (28.6%)	1.53 (1.13–2.06)	1.0	1.49 (1.08–2.06)	
Hypertension	593 (24.6%)	1.38 (1.06–1.80)	1.0	1.78 (1.34–2.36)	0.547
No hypertension	170 (16.5%)	1.44 (0.93–2.25)	1.0	1.55 (0.96–2.53)	
Diabetes mellitus	286 (43.2%)	1.46 (0.95–2.23)	1.0	2.02 (1.30–3.14)	0.397
No diabetes mellitus	477 (17.1%)	1.37 (1.05–1.79)	1.0	1.61 (1.21–2.16)	
Prior cardiovascular disease	141 (38.3%)	1.64 (0.71–3.77)	1.0	1.89 (0.76–4.69)	0.057
No prior cardiovascular disease	622 (20.2%)	1.42 (1.04–1.95)	1.0	1.38 (0.98–1.94)	
Hyperlipidemia	147 (24.6%)	0.80 (0.46–1.40)	1.0	2.77 (1.58–4.88)	0.034
No hyperlipidemia	616 (21.6%)	1.56 (1.22–2.01)	1.0	1.54 (1.17–2.02)	

*All analyses adjusted for age, sex, body mass index, current smoker, current alcohol use, systolic blood pressure, comorbidities, medication use, and lab measurements.*

**TABLE 6:** Association of creatine kinase with cardiovascular mortality in subgroups.

	Creatine kinase				
	Event rates	Low (<111 IU/L)	Moderate (111–179 IU/L)	High (>179 IU/L)	P-interaction
**Cardiovascular mortality**					
<65 years	246 (8.6%)	1.41 (0.98–2.01)	1.0	1.41 (0.96–2.08)	0.678
≥65 years	138 (23.7%)	1.46 (0.84–2.52)	1.0	1.60 (0.88–2.90)	
Male	157 (8.7%)	2.03 (1.22–3.38)	1.0	2.38 (1.40–4.07)	0.078
Female	227 (13.8%)	1.23 (0.85–1.78)	1.0	1.06 (0.71–1.60)	
Hypertension	308 (12.8%)	1.40 (1.01–1.96)	1.0	1.45 (1.01–2.08)	0.804
No hypertension	76 (7.4%)	1.58 (0.84–2.98)	1.0	1.38 (0.68–2.80)	
Diabetes mellitus	132 (19.9%)	1.50 (0.89–2.78)	1.0	1.58 (0.89–2.78)	0.478
No diabetes mellitus	252 (9.1%)	1.42 (1.00–2.02)	1.0	1.38 (0.94–2.04)	
Prior cardiovascular disease	62 (16.8%)	1.65 (1.03–1.94)	1.0	1.85 (1.01–1.96)	0.180
No prior cardiovascular disease	322 (10.5%)	1.42 (0.72–3.79)	1.0	1.39 (0.76–4.55)	
Hyperlipidemia	50 (8.4%)	0.80 (0.34–1.87)	1.0	2.04 (0.89–4.67)	0.023
No hyperlipidemia	334 (11.7%)	1.56 (1.14–2.14)	1.0	1.33 (0.94–1.88)	

*All analyses adjusted for age, sex, body mass index, current smoker, current alcohol use, systolic blood pressure, comorbidities, medication use, and lab measurements.*

### Sensitivity Analysis

The exclusion of patients with prior cardiovascular disease or those who died in the first 2 years of follow-up did not materially affect the results from the creatine kinase analyses (Tables 3, 4).

When considering a transfer to hemodialysis, receiving renal transplantation, transfer to other centers, and also loss of follow-up as competing risks for all-cause mortality, we found that compared with the moderate group, the high and low groups were associated with 1.32 (95% CI, 1.05–1.68; *E*-value = 1.97) and 1.54 (95% CI, 1.09–2.19; *E*-value = 2.45) times of risk of all-cause mortality, respectively (Supplementary Table 1).

## Discussion

In this large, multi-center, retrospective cohort study, we investigated the association between serum creatine kinase and mortality in patients with PD. The lowest all-cause and cardiovascular mortality risks were seen among patients with serum creatine between 111 and 179 IU/L. Higher and lower creatine kinase levels were associated with increased risks, resulting in a U-shaped association curve. Although our findings were inconsistent with those previous findings (10–13), the association between creatine kinase and mortality in our study may be more plausible in clinical practice.

More excellent creatine kinase activity is thought to promote vascular contractility and retain sodium (6, 19). Individuals with high creatine kinase activity are at a greater risk of developing hypertension, with more excellent resistance against blood pressure-lowering therapy (6). Previous studies showed elevated serum creatine kinase levels were associated with increased mortality in patients with different comorbid conditions (10, 12, 13). A previous study reported high creatine kinase levels in patients on dialysis compared with the healthy controls, and hemodialysis did not seem to contribute to the creatine kinase elevation. Plus, post-dialysis creatine kinase values were lower (albeit not significant) when compared with pre-dialysis values (14). The fall of post-dialysis creatine kinase values was possibly due to a change of blood PH during dialysis and the use of a dialysis bath containing a significant quantity of sodium acetate, which can inhibit the activity of creatine kinase (20). Therefore, to eliminate the effect of dialysis on creatine kinase, we examined the association between creatine kinase before the first PD procedure and death during the follow-up period. We found a U-shaped association between creatine kinase and mortality. Even after adjusting for confounding factors or sensitivity analyses, the results remained robust. Additional subgroup analyses found an interaction between no hyperlipidemia and serum creatine kinase. In patients with hyperlipidemia, low levels of creatine kinase were associated with a high risk of all-cause and cardiovascular mortality, but no significant association in those with hyperlipidemia. Medication for hyperlipidemia that may lead to serum creatine kinase elevation (21, 22). Nonetheless, how no hyperlipidemia modified the association between low creatine kinase levels and mortality is inconclusive.

Although the previous study reported elevated creatine kinase in dialysis patients (14), the association between creatine kinase and prognosis of patients on dialysis remained unknown. Due to the high presence of comorbidities, such as diabetes mellitus, hypertension, prior cardiovascular disease, and hyperlipidemia, which may influence the association between creatine kinase and mortality in dialysis patients, it is difficult to speculate on the association. In our study, we found a specific association in CAPD patients. Data analyzed that lower or higher serum creatine kinase levels were associated with increased all-cause and cardiovascular mortality risks. A previous study found that lower serum creatine kinase levels may reflect poor nutritional status (5). As we all know, poor nutritional status was associated with an increased risk of all-cause mortality (23). Early publications estimated that 40–66 percent of patients with PD in the United States are malnourished (24–26). Meanwhile, individuals with higher serum creatine kinase levels were at greater risk of developing hypertension, with more excellent resistance against blood pressure-lowering therapy (6, 8). Therefore, based on these aforementioned findings, our findings may be more plausible in the clinical practice setting.

To date, there was one study reporting the association between serum creatine kinase and mortality in non-dialysis CKD patients, which reported that a low level of serum creatine kinase was associated with an increased risk of death (5). This study only included patients with CKD with a median eGFR of 40 ml/min × 1.73 m^2^, and excluded those with eGFR <15 ml/min × 1.73 m^2^. Participants in this study were artificially divided into three groups, affecting the association between creatine kinase and death. Plus, this study included participants at high cardiovascular risk and was vulnerable to biases from reverse causation. Reverse causation may occur when patients with prior cardiovascular disease or increased cardiovascular risk reduce their exercise magnitude due to illness or medical recommendations, reducing serum creatine kinase levels (27). Our study excluded patients with prior cardiovascular disease or those who died in the first 2 years of follow-up did not materially alter our findings. Nonetheless, we acknowledged that reverse causation cannot be completely ruled out. Therefore, a large prospective study is required to verify our results.

Strengths included a large number of patients, high completeness of data, and the availability of detailed covariates to adjust for a broad range of potential confounders. Our study also had several limitations, and findings should be interpreted with these in mind. First, as with all observational studies, a potential limitation of our study was the possibility of residual confounding from unmeasured variables. However, the *E*-value analysis showed that a confounder effect would need to be markedly large to alter the direction of association in the multivariable models. For example, even a strong confounder effect (HR ≥ 2.83) would need to be considerably imbalanced between the high and moderate creatine kinase categories to result in an adjusted hazards ratio below 1.0. Weaker confounders could not do so. Second, the single serum creatine kinase measurement at baseline may have underestimated the association between serum creatine kinase levels and mortality because of the regression dilution bias (28). However, regression dilution bias may lead to over-adjustment (29). Third, missing values were replaced by the most recent available deals, not using multiple imputations. Although multiple imputations can randomly fill these missing values, the most recent available values may more appropriately present a patient’s clinical status. Fourth, we had not excluded patients taking drugs that might affect serum creatine kinase and did not evaluate the association between serum creatine kinase and non-fatal cardiovascular events.

In conclusion, a serum creatine kinase before the start of PD between 111 and 179 IU/L was associated with a lower risk of all-cause and cardiovascular mortality than a higher or lower level of creatine kinase, resulting in a U-shaped association curve. Prospective analyses on these associations are needed for causal inferences and to decide whether creatine kinase could serve as a new, clinically helpful biomarker for prognosis in patients with PD.

## Data Availability Statement

The raw data supporting the conclusions of this article will be made available by the authors, without undue reservation.

## Ethics Statement

The studies involving human participants were reviewed and approved by the Ethics Committee of the First Affiliated Hospital of Zhengzhou University, the Ethics Committee of the First Affiliated Hospital of Nanchang University, the Ethics Committee of Jiujiang No.1 People’s Hospital, the Ethics Committee of Zhujiang Hospital of Southern Medical University, and the Ethics Committee of the Second Affiliated Hospital of Guangzhou Medical University. Written informed consent for participation was not required for this study in accordance with the national legislation and the institutional requirements.

## Author Contributions

XFW and LZ contributed to the conception, interpretation of data, and drafted the work. XYW, XZ, XF, and FP contributed to the acquisition, analysis, and interpretation of data. NW and YW contributed to the conception and design of the work. XFW and JW contributed to the conception, design of the work, and revised it. All authors read and approved the manuscript and met the criteria for authorship.

## Conflict of Interest

The authors declare that the research was conducted in the absence of any commercial or financial relationships that could be construed as a potential conflict of interest.

## Publisher’s Note

All claims expressed in this article are solely those of the authors and do not necessarily represent those of their affiliated organizations, or those of the publisher, the editors and the reviewers. Any product that may be evaluated in this article, or claim that may be made by its manufacturer, is not guaranteed or endorsed by the publisher.
