# Supplementary material for: Creatine Kinase and Mortality in Peritoneal Dialysis
Source: Front Cardiovasc Med. 2022 May 10;9:855891. doi: 10.3389/fcvm.2022.855891 (PMC9127078; doi:10.3389/fcvm.2022.855891)
Supplement: Supplementary file 1 [file Data_Sheet_1.docx]

**Creatine Kinase and Mortality in Peritoneal Dialysis**

**Supplementary Appendix**

**Materials & Methods**

**Study Design and Participants**

***Peritoneal dialysis centers of five tertiary hospitals***

The First Affiliated Hospital of Zhengzhou University, Zhengzhou, China.

The First Affiliated Hospital of Nanchang University, Nanchang, China.

Jiujiang No. 1 People’s Hospital, Jiujiang, China.

Zhujiang Hospital of Southern Medical University, Guangzhou, China.

The Second Affiliated Hospital of Guangzhou Medical University, Guangzhou, China.

***Data Collection***

Patients were advised to initiate peritoneal dialysis (PD) with professional and clinical evaluation from nephrologists. In all patients, thorough medical records were reviewed by trained nurses in each dialysis center at study entry. In China, the patient must receive the first dialysis in the hospital, suggesting most of the patient’s data can be obtained within one week before the first dialysis. Thus, we defined baseline as one week (5.3±1.2 days) before the first continuous ambulatory peritoneal dialysis (CAPD). All laboratory parameters from fasting blood samples were measured in each tertiary hospital's laboratory department.

***Dialysis procedure***

All patients received CAPD treatment. Conventional dialysis solutions (Dianeal 1.5%, 2.5%, or 4.25% dextrose; Baxter Healthcare, Guangzhou, China), Y sets, and twin bag systems were used in all CAPD patients. No patients received automated PD.

***Follow-up***

There was no exposure to all patients with any intervention. Patients needed to return to each center at least quarterly for an overall medical assessment. The trained nurses conducted monthly face-to-face interviews or monthly telephone interviews to assess their general condition and related medications.

***Outcome measurements***

We determined death causes based on medical files of admission. If patients died out of hospitals, we determined death causes according to interviewing with family members by telephone to acknowledge death's circumstances, combining with information from medical records of peritoneal dialysis centers.

***Definitions***

Cardiovascular mortality included death associated with an acute myocardial ischemic event, heart failure, hemorrhagic or thromboembolic stroke, malignant arrhythmia, and sudden cardiac death, based on the International Classification of Diseases Clinical Modification, 9th Revision. Sudden cardiac death is defined as unexpected, nontraumatic death occurring within 1hour of the onset of new or worsening symptoms (witnessed arrest) or, if unwitnessed, within 24 hours of last being seen alive^1^. Hypertension was defined as systolic blood pressure > 140mmHg, diastolic blood pressure > 90 mmHg, or the use of antihypertensive medications. Diabetes mellitus was defined as a history of diabetes mellitus. Hyperlipidemia was defined as (1) serum cholesterol levels ≥4.7 mmol/L, (2) triglyceride levels ≥2.3 mmol/L, or (3) low density lipoprotein levels ≥4.1 mmol/L^2^. Patients who meet one of these three items are defined as having hyperlipidemia. Current smoking was defined as at least one cigarette a day, and current alcohol consumption was defined as > 20 g of ethanol a day^3^. Chronic obstructive pulmonary disease is characterized by persistent respiratory symptoms and progressive airflow obstruction^4^. Gastrointestinal bleeding includes upper and lower gastrointestinal bleeding^5^. The Chronic Kidney Disease Epidemiology Collaboration equation was used to calculate eGFR^6^.

***Missing data***

In China, the patient must receive the first dialysis procedure in the hospital. To obtain missing patients’ demographic characteristics, comorbid conditions, medication use, and laboratory variables, we had checked the medical records of receiving the first PD procedure. Thus, missing data for serum creatine kinase (n=97) or any other explanatory variables (n=143) at the start of PD were replaced by the most recent available values by checking patients’ medical records of receiving the first PD procedure.

**Table S1. Association of creatine kinase with mortality using competing risk model.**

|  | Creatine kinase | | |
| --- | --- | --- | --- |
|  | Low (< 111 IU/L) | Moderate (111-179 IU/L) | High (> 179 IU/L) |
| Hazards ratio (95%CI) | 1.32 (1.05-1.68) | 1.0 | 1.54 (1.09-2.19) |

Transfer to hemodialysis, receiving renal transplantation, transfer to other centers, and loss of follow-up were competing risks for all-cause mortality.

All analyses adjusted for age, sex, body mass index, current smoker, current alcohol use, systolic blood pressure, comorbidities, medication use, and laboratory measurements, using the Gray test.

***Sample size***

Due to a retrospective cohort design, we had collected as many patients’ data as possible and did not pre-estimate the sample size for our study. We conducted a posthoc power analysis based on the available sample size using power analysis based on the Cox regression model. An effect size of 3446 or more was considered to be clinically significant. A post hoc statistical power calculation confirmed that the sample size available for this study provided more than 90% power to detect such clinically significant differences.

**Reference**

1. European Heart Rhythm A, Heart Rhythm S, Zipes DP, et al. ACC/AHA/ESC 2006 guidelines for management of patients with ventricular arrhythmias and the prevention of sudden cardiac death: a report of the American College of Cardiology/American Heart Association Task Force and the European Society of Cardiology Committee for Practice Guidelines (Writing Committee to Develop Guidelines for Management of Patients With Ventricular Arrhythmias and the Prevention of Sudden Cardiac Death). *J Am Coll Cardiol.* 2006;48(5):e247-346.

2. Joint committee issued Chinese guideline for the management of dyslipidemia in a. [2016 Chinese guideline for the management of dyslipidemia in adults]. *Zhonghua Xin Xue Guan Bing Za Zhi.* 2016;44(10):833-853.

3. Tu W, Wu J, Jian G, et al. Asymptomatic hyperuricemia and incident stroke in elderly Chinese patients without comorbidities. *Eur J Clin Nutr.* 2019;73(10):1392-1402.

4. Labaki WW, Rosenberg SR. Chronic Obstructive Pulmonary Disease. *Ann Intern Med.* 2020;173(3):ITC17-ITC32.

5. Marek TA. Gastrointestinal bleeding. *Endoscopy.* 2011;43(11):971-977.

6. Zhang L, Wang F, Wang L, et al. Prevalence of chronic kidney disease in China: a cross-sectional survey. *Lancet.* 2012;379(9818):815-822.
